# Voltammetric estimation of residual nitroxynil in food products using carbon paste electrode

**DOI:** 10.1038/s41598-022-18305-6

**Published:** 2022-08-22

**Authors:** Mohamed M. Salim, Sally Ashraf, Heba M. Hashem, Fathalla Belal

**Affiliations:** 1 Pharmaceutical Analytical Chemistry Department, Faculty of Pharmacy, Mansoura University, P.O. Box 35516, Mansoura, Egypt; 2 Pharmaceutical Chemistry Department, Faculty of Pharmacy, Horus University-Egypt, P.O. Box 34518, New Damietta, Egypt

**Keywords:** Analytical chemistry, Electrochemistry

## Abstract

A simple and sensitive voltammetric method was developed and validated for the recognition of the veterinary drug nitroxynil (NTX). The method is based on studying its voltammetric behavior at a carbon paste electrode. Square wave voltammetry (SWV) was successfully applied in this study. The anodic peak current obtained was a linear function of NTX concentration in Britton Robinson buffer of pH 3 over the range of 3.9 × 10^–6^–1.0 × 10^−4^ M with lower detection and quantitation limits of 3.1 × 10^–7^ and 9.4 × 10^–7^ M, respectively. The proposed method was first applied to the assessment of the drug in commercial vials. The method was further used to monitor the residual amounts of the drug in bovine meat, kidney, fat, and milk samples. The results obtained were favourably compared with those given by reference method. The interference likely to be introduced by co-administered drugs was evaluated. The electrode reaction was elucidated, and electron transfer kinetics were studied.

## Introduction

Veterinary therapies and feed supplements are extensively utilized in animal farming to overcome and treat diseases, protect animals’ health, and promote their growth. Many of these compounds, including antiparasitic, anthelmintic, and antiprotozoal drugs, are administered to avoid economic losses caused by diseases in animal husbandry processes and progress in many countries, raise individual animals, and raise individual animals' herd performance^1^.

**Figure 1:**
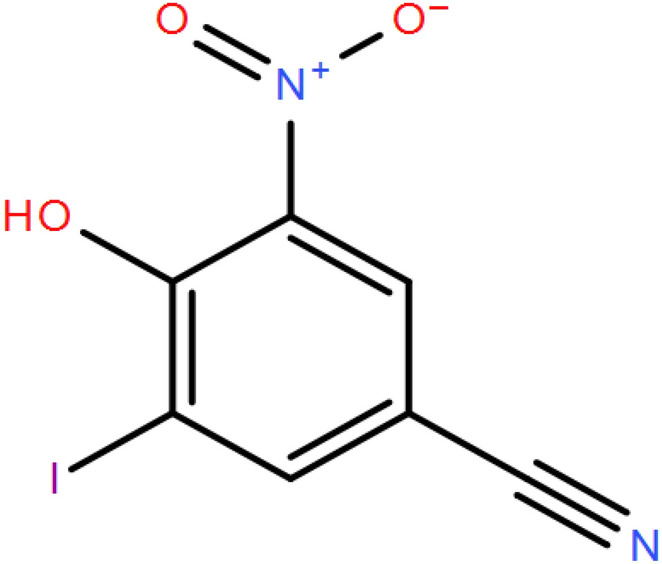
Chemical structure of nitroxynil.

Nitroxynil (4-hydroxy-3-iodo-5-nitrobenzenonitrile, NTX, Fig. 1) is an anthelmintic veterinary drug commonly used in the prophylaxis and dealing withhepatic distomatosis. It controls fascioliasis,the cause of Fasciola hepatica in cattle and sheep, and exhibits antiparasitic effects^2,3^. Compared with other fasciolicides, NTX displays higher activity against immature and adult liver flukes^4,5^. Drug residues in food products have deep concern because they may be a reason for allergic reactions in sensitive individuals or engage in the growth of antibiotic-reluctant pathogenic bacterial strains^6,7^.

Nitroxynil minimum regulatory limits (MRLs) were established according to European Commission Regulations (ECR) No. 997/1999 for the validation of analytical methods in bovine and ovine muscles, fat, liver, and kidney to be 400, 200, 20, and 400 μg/kg, respectively^8^. Therefore, monitoring MRL concentrations of NTX in different food samples is highly recommended. The reported methods and techniques for determining residual levels of NTX in food samples include high-performance liquid chromatography (HPLC)^9–11^, gas chromatography^12–14^, Spectrofluorometry^15^, immunobiosensor^16^, immunochromatographic strip^17^ polarography^18–22^, and voltammetry^23,24^. Most of the reported analytical methods have shortcomings in quantifying residual limits of NTX or need high-cost techniques. Chromatographic methods have long been utilized for this destination; their operational process needs skilled operators or high-cost instrumentation^25^. Also, polarographic methods were restrained by the disadvantages of utilizing mercury electrode, which is environmentally toxic, while the others have utilized costly electrodes. Thus, developing a simple, cost-effective, and sensitive method is highly needed for quantifying NTX in its pharmaceutical forms and food samples. In this regard, and to achieve this purpose, square wave voltammetry (SWV) was adopted for the determination of NTX in food samples based on the use of carbon paste electrode (CPE).

Carbon electrodes, particularly carbon paste electrodes, are extensively used in electrochemical investigations because of their low background current, extensive potential windows, chemical inertness, low cost, fast response, and more environmentally friendly techniques^26–29^. Also, they provide suitability for recognizing several organic and biological compounds^30–33^. CPE is widely applicable in electroanalysis and electrochemical studies because of its easy fabrication, ease of miniaturization, and simplicity of surface renewal process^34–37^.

Under the aforementioned umbrella, this study was undertaken for a simple, sensitive, and cost-effective recognition of NTX in its parenteral pharmaceutical formulations and residual determination in food products could be accomplished. The developed method could be exploited to determine the drug within MRLs values stated by the European Commission Regulations No 997/1999^8^. The proposed method was validated according to the International Conference on Harmonization (ICH) guidelines^38^.

## Experimental

### Materials and methods

Nitroxynil ((B.N.0000124001) was kindly provided by Natco. Lab. Chemical Reagents Co., Cairo, Egypt; its purity was certified to be 100.12%). PIONIX 25% vials [B.N. 140429] each 100-mL vial contains 25 gm NTX (Allam Pharmaceutical Industries Co., Cairo, Egypt). Graphite powder (size, < 20 μm, synthetic, Molecular Weight:12.01 g/mol, CAS Number: 7782-42-5, Merck, Darmstadt, Germany) and paraffin oil (viscous liquid, density 0.827–0.890 g/mL at 20 °C, CAS Number: 8012-95-1, Merck, Darmstadt, Germany).

Food products: bovine meat, kidney, fat, and milk samples were purchased from the local shop. Britton-Robinson buffer (BRb) was prepared as a mixture of phosphoric acid, acetic acid, and boric acid of identical concentrations (0.04 M) each, and adjusted to pH with definite volumes of 0.4 M NaOH to prepare a wide pH range (2.2–12). Methanol (HPLC grade) was purchased from Sigma-Aldrich. Throughout the study, deionized water was used to prepare all solutions.

### Equipments

Voltammetric measurements were carried out using a Metrohm Voltamograph (884 Professional VA, Utrecht, Netherlands). One compartment cell with a three-electrodes set-up including carbon paste electrode coupled with a reference electrode (Ag/AgCl/KCl (3 M) and Pt auxiliary electrode. The electrochemical process was performed at the ambient temperature of 25 °C. The stored results were retrieved using VIVA 2.1 software. The pH measurements were conducted and regulated by a pH Meter (Jenway 3505 Instruments, UK). A vortex mixer (Zx4, VELP ScientificaSrl, Italy) was used for blending the solutionsand centrifuge (Model Sigma 2-16P, Germany). A glass homogenizer (glass-col, 099ck424, Korea) was utilized for sample homogenization.

### Procedures

#### Preparation of working electrode (CPE)

CPE was prepared by mixing 0.5 g of graphite powder and three drops (0.3 mL) of paraffin oil in a small agate mortar. The homogenized paste was introduced into an insulin syringe with a cross-section of 1.34 mm and flattened on a filter paper to obtain a polished appearance. For electrochemical measurements, a copper wire was touched with the carbon paste from the syringe end side and used to connect with the electrochemical device as a working electrode. After every measurement, the paste was neatly removed before pressing a new section into the electrode.

#### Preparation of standard and working solutions

##### Electrochemical measurements

The stock solution of NTX (1.0 × 10^–3^ M) was prepared by dissolving 14.0 mg of the pure drug in 50 mL of methanol in a measuring flask, then sonicating until complete dissolution. Various serial dilutions were performed to get different concentrations of NTX (1.0 × 10^−4^–1.0 × 10^−6^ M) by completing aliquots of standard solution with BRb of pH 3. All prepared solutions were kept at 4 °C and protected from light**.** The standard solution is stable for at least 2 weeks.

Cyclic and square-wave voltammetric modes were utilized for the implementation of the electrochemical studies of NTX at the CPE surface. The optimization of SWV parameters was done at different values for potential step (3–20 mV), frequency (5–30 Hz), pulse amplitude (10–60 mV), and various scan rates (20–300 mV s^−1^). NTX monitoring was achieved by adopting the SWV mode at the following optimized parameters: a potential step of 20 mV, frequency of 20 Hz, pulse amplitude of 60 mV, and a scan rate of 100 mV s^−1^. All scans were carried out in the positive direction with an applied potential range of + 20 to + 1600 mV at ambient temperature. The variation of pH over the range of 2.5–9 was applied to investigate its effect on the cyclic voltammetric behavior of the drug.

#### Construction of the calibration curve

Different concentrations of NTX (3.9 × 10^–6^–1.0 × 10^−4^ M) were prepared by transferring aliquots of NTX standard solution into a series of 25 mL volumetric flasks. BRb of pH 3 was used to complete the volumes to the mark. The solutions were transferred quantitatively into the micro-electrolysis vessel and deoxygenated with nitrogen for 5 min. The anodic peak current (Ip) was measured in the positive direction and plotted versus drug concentration (M), then the regression equation was derived. The nominal concentration of NTX was calculated using the regression equation.

#### Analysis of NTX in parenteral solutions

Five PIONIX [Nitroxynil 25%] vials were mixed. An aliquot (0.11 mL) of the mixed solution was transferred into a 10 mL volumetric flask and completed to volume with methanol to prepare a stock solution of concentration of 1.0 × 10^–2^ M of NTX. Further dilutions were done with BRb (pH 3) to give a working solution equivalent to 3.9 × 10^–6^–1.0 × 10^−4^ M.

#### Application to bovine meat, kidney, and fat samples

NTX was spiked to bovine meat, kidney, and fat samples. Each sample (5 g) was accurately weighed and homogenized with NTX and methanol at 5000 rpm for 5 min. The homogenate was sonicated for 15 min then centrifuged at 3000 rpm for another 5 min. 2 mL of the supernatant of all samples was transferred into 10 mL volumetric flasks and completed to the mark with methanol and filtered through 0.45 μm syringe filters. Then aliquots of NTX working standard solutions equivalent to (1.7 × 10^–6^–6.8 × 10^−4^ M) were transferred into a series of 25 mL volumetric flasks and completed to the mark with BRb of pH 3. The linearity was investigated by plotting the peak current (Ip) versus drug amount (M).

#### Preparation of bovine milk samples

Milk sample (5 mL) was transferred into a 25 mL volumetric flask, spiked with aliquots of NTX standard solution then vortex mixed. Protein precipitation was carried out by adding 5 mL of 1 N HCL^39^, and the supernatant was filtered through 0.45 μm syringe filters and transferred to a 25 mL volumetric flask. The volume was completed to the mark with methanol. Aliquot of the previously filtered supernatant was then transferred to the voltammetric cell, completed to the mark with BRb of pH 3, and the SW voltammograms were recorded. Then the % recoveries were calculated.

## Results and discussion

### Electrochemical investigation of NTX at CPE

#### Effect of pH

**Figure 2:**
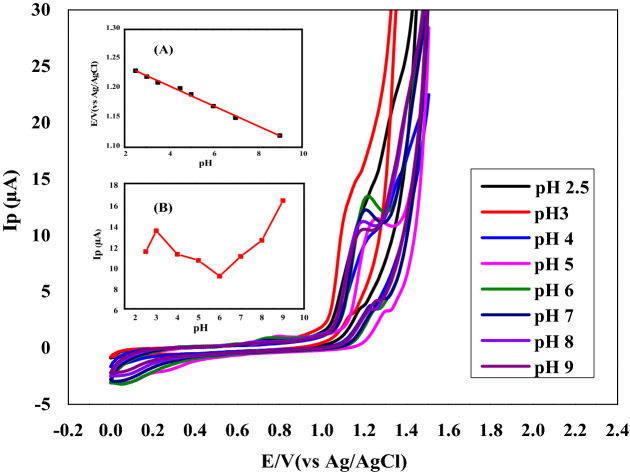
Cyclic voltammographs of 1.18 × 10^−5^ M Nitroxynil at different pH values at carbon paste electrode using scan rate of 100 mV s^−1^. Inset A: relation between anodic peak potential and pH recorded at carbon paste electrode. Inset B: relation between current and pH.

Preliminary studies using cyclic voltammetry display the behavior of irreversible oxidation of NTX at CPE. Figure 2 demonstrates typical cyclic Voltammograms of 1.18 × 10^−5^ M of NTX in BRb at different pHs over the range of 2.5–9, at a sweep rate of 100 mV/s at CPE. BRb was chosen as the supporting electrolyte in all measurements^24^. Figure 2A was plotted between peak potential Ep (V) against different pHs, and as shown, the peak potentials are shifted to less positive values by increasing the pH of the tested solutions. These results signify that the oxidation of NTX is pH-dependent, and it exhibited a linear regression plot obeying the following equation:

1 $${\text{Ep }}\left( {\text{V}} \right) = 1.2723- 0.017{\text{ pH}} \quad ({\text{r}}^{2} = 0.995)$$

Implying that protons and electrons are directly involved in the oxidation process. The highest anodic current for NTX was obtained at pH 3. Considerable lowering in Ip values with increasing pH values till pH 6 was noticed, but a significant increase at higher pHs was observed (Fig. 2B). Thus pH 3 was chosen as the most suitable one for further investigations.

#### Effect of scan rate

**Figure 3:**
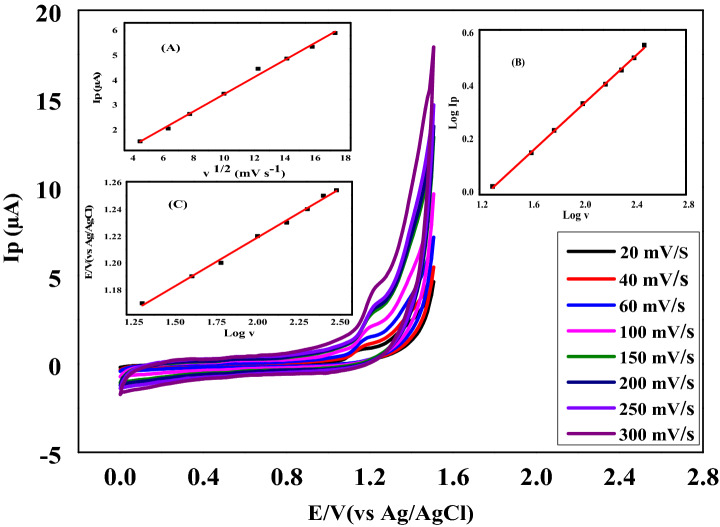
Cyclic voltammograms of 1.96 × 10^−5^ M of Nitroxynil in BRb (pH 3) at different scan rates (20.0–300.0 mV s^−1^) inset (A): relation between log anodic peak current and the square root of scan rate, inset (B): relation between log anodic peak current and log scan rate, inset (C): relation between peak potential and log scan rate.

The electrochemical behavior of NTX was investigated using various scan rates (20–300 mV s^−1^) for 1.96 × 10^−5^ M of NTX in BRb of pH 3. The electrochemical mechanism could be explained by plotting the relationship between peak current and scan rates as shown in Fig. 3. As the scan rate increased, the oxidation peak current increased; this process suggested the kinetics of redox reaction sites of NTX on CPE^40^. Diffusion or adsorption-controlled mechanisms were suggested on CPE in NTX determination^41^. Fig. 3A shows high linearity (r^2^ = 0.994) of the relationship between the peak current (Ipa) and the square root of the scan rate (v^1/2^) as expressed in Eq. (2); this specifies that the oxidation process was controlled by diffusion phenomenon^42^.

2 $${\text{Ip }}\left( {\upmu {\text{A}}} \right) = 0.344 \text{v}^{1/2} \left( {{\text{mV}}\;{\text{s}}^{ - 1} } \right) - 0.01$$

Furthermore, a linear relationship ((r^2^ = 0.999) was exhibited from the plot of logarithm of peak current versus scan rates values (Fig. 3B) as seen in the following equation:

3 $${\text{Log Ip }} = - 0.567 + 0.447 \, \log \upnu$$

The slope value (0.447) is close to 0.5, confirming that the proposed mechanism of the electrochemical process on CPE was controlled by diffusion of electroactive species. As shown in Fig. 3C, the electrochemical oxidation peak potential (Ep) also relied on scan rate values. As the sweep rates increased, the potentials were shifted to more positive values, and the derived Eq. (4) exhibited good linearity (r^2^ = 0.994).

4 $${\text{Ep }}\left( {\text{V}} \right) = 1.073 + 0.072 \, \log \upnu$$

The kinetic parameters of the electron-transfer process were evaluated adopting Laviron’s theory for the irreversible process, and exhibited the number of electrons relocated as seen in the following equation^43^:

5 $${\text{E }} = {\text{ Eo }} + 2.303{\text{ RT}}/\upalpha {\text{nF }}\left[ {\log {\text{RTKo}}\upalpha {\text{nF}}} \right] + 2.303{\text{ RT}}/\upalpha {\text{nF }}\left( {\log \, \upnu } \right)$$

where, R is the gas constant (8.314 J K mol^−1^), T is the absolute temperature, F is the Faraday constant (96,485 Coulomb. mol^−1^), α is the electron transfer coefficient, and n is the number of the transferred electrons.

The slope from the linear relationship between potential against log scan rate was used to calculate αn. Using this method, the slope value is 0.113, from which αn value was calculated to be 0.523. As α was assumed to be 0.5 for all irreversible electron transfer via redox reactions, n was found to be 1.04, approximating referring to one electron transfer in the oxidation of NTX on CPE^44^.

### Method validation

According to ICH guidelines^38^ and The U.S. Food and Drug Administration (FDA) recommendations and based on analytical procedures validation and documentations^45^, the proposed method was validated. The analytical behavior of the proposed electrochemical sensor for NTX was studied by analyzing three batches (3 replicates each) of the standard solutions to show the linear range, detection and quantification limits, and precision (standard deviation), accuracy (trueness).

#### Limits of detection and quantification and method linearity

LOD and LOQ were calculated to be 3.1 × 10^–7^ and 9.4 × 10^–7^ M, respectively, using to the following equations^38^:

6 $${\text{LOD }} = 3.3 \upsigma/{\text{S}}$$

7 $${\text{LOQ }} = 10 \upsigma/{\text{S}}$$

where “σ” is the standard deviation of intercept and “S” is the slope of the calibration curve.

**Figure 4:**
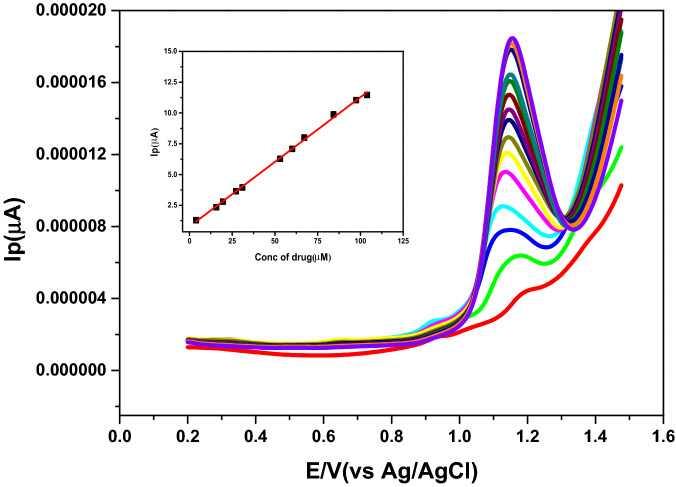
Square-wave voltammograms of different concentrations of Nitroxynil (3.9 × 10^–6^–1.0 × 10^−4^ M) in BRb (pH 3) at scan rate100 mV s^−1^, using carbon paste electrode. insert: plots of Ip *vs*. Nitroxynil concentrations.

Figure 4 showed a wide linearity range of the method that exhibited over the range of 3.9 × 10^–6^–1.0 × 10^−4^ M. Table 1 summarized the calibration data and the corresponding validation parameters.

**Table 1:** Validation parameters of the proposed SWV method for determination of NTX .

Validation parameter	Value
Linearity range (M)	3.9 × 10^–6^–1.0 × 10^−4^
Intercept (a) (μA)	10.74
Slope (b) (μA/M)	6.25
Correlation coefficient (r^2^)	0.999
S.D. of residuals (S_y/x_)	1.58
S.D. of intercept (S_a_)	0.59
S.D. of slope (S_b_)	0.06
LOD (M)	3.1 × 10^–7^
LOQ (M)	9.4 × 10^–7^

In comparison with the other reported electrochemical techniques, Table 2 illustrates the analytical features of the reported ones and the proposed approach for NTX determination.

**Table 2:** Comparison of the analytical features of different electrodes used for determination of NTX.

Electrode	Method	Optimum pH	LOD/M	Linear Range/M	Ref
Dropping mercury electrode	–	6.0	–	0.1 × 10^–3^–0.5 × 10^–3^	^22^
Mercury electrode	DC-polarography	6.0	3 × 10^–5^	3 × 10^–5^–2.5 × 10^–4^	^18^
	Differential-pulse adsorptive stripping voltammetry	6.0–7.0	1.31 × 10^–8^	–	
	Square-wave adsorptive stripping voltammetry	6.0–7.0	8.4 × 10^–10^	–	
Glassy carbon electrode (GCE)	SWV	2.0	0.28 × 10^–6^	3.49 × 10^–6^–47.62 × 10^–6^	^24^
GCE modified with SWCNT	SWV	2.0	0.36 × 10^–6^	2.00 × 10^–6^–99.10 × 10^–6^	^23^
GCE modified with MWCNT			0.16 × 10^–6^	0.99 × 10^–6^–90.91 × 10^–6^	
GCE modified with Graphene functionalized using carboxylic groups			0.11 × 10^–6^	2.99 × 10^–6^–65.42 × 10^–6^	
GCE modified with carbon nanohorns (CNHs)			0.34 × 10^–6^	2.00 × 10^–6^–90.91 × 10^–6^	
Carbon paste electrode (CPE)	SWV	3.0	3.1 × 10^–7^	3.9 × 10^–6^–1.0 × 10^−4^	This work

#### Accuracy and precision

To ensure the method's accuracy, triplicate analysis of three different concentrations were measured. The mean percentage recoveries were calculated as shown in Table 3. The proposed method's accuracy was statistically evaluated by comparing the results attained by the proposed method with those given by the official method^46^ using the t-test and F-test. No significant difference was found between them. The official method depends on measuring the UV absorbance of an aqueous alkaline solution of the drug at 271 nm, both in pure form and injections.

**Table 3:** Statistical analysis of the results obtained by the proposed and official method for determination of NTX.

Parameters	Proposed method			Official method^46^
	Amount taken (× 10^–6^ M)	Amount found (× 10^–6^ M)	% found^a^	
	1.99	1.94	97.64	101.15
	3.98	4.06	102.09	99.03
	7.93	7.91	99.77	100.18
	13.02	13.01	99.95	
	14.19	14.19	99.99	
Mean ± S.D			99.88 ± 1.57	100.12 ± 1.06
t^b^	0.20 (2.77)^b^			
F^b^	4.39 (19.00)^b^			

^a^Average of 3 replicate determinations.

^b^The values between parentheses are the tabulated values of *t* and *F* at *P* = 0.05^47^.

Inter-day precision was assessed by measuring three different concentrations, in triplicates, in three consecutive assays. Nevertheless, intra-day precision was evaluated for three concentrations in triplicates at the same assay. The relative standard deviations were less than 2%, as shown in Table 4.

**Table 4:** Inter-day and intra-day precision data using the proposed method.

Parameters	Inter-day Conc. taken (× 10^−5^ M)			Intra-day Conc. taken (× 10^−5^ M)		
	8.42	5.3	1.96	8.42	5.3	1.96
% Found	97.84	102.09	99.58	99.51	101.21	99.81
	97.85	102.14	99.58	99.71	100.81	100.11
	98.04	101.91	99.62	100.11	101.01	99.70
(${\overline{\text{x}}}$)	97.91	102.05	99.59	99.77	101.01	99.87
± S.D	0.12	0.09	0.02	0.31	0.16	0.21
%RSD	0.12	0.09	0.02	0.31	0.16	0.21
%Error	0.07%	0.05%	0.01%	0.18%	0.09%	0.12%

N.B. Each result is the average of three separate determinations.

#### Method robustness

The robustness was tested by deliberated slight variations in the experimental conditions to exhibit unbiased results. The considered variables involved minor changes in pH (3.0 ± 0.4) and the applied time before each measurement (20 s ± 3 s). During the experimental procedure, these slight changes had no impact on the peak current strength, signifying the reliability of the applied method during the regular procedure.

#### Specificity

The specificity of the method was illustrated by exploring the effect of biological tissue matrices in bovine meat, kidney, milk, and fat samples as well as common excepients in veterinary formulations. Any of these did not affect the application of the suggested method for determination of NTX as revealed by the high percentage recoveries as illustrated in Tables 5 and 6.

**Table 5:** Determination of NTX in its parenteral injection using the proposed and reference method.

PIONIX 25% vials NTX (25 gm/100 mL)	Proposed SWV Method			Official method^46^
	Conc. taken (× 10^−6^ M)	Conc. found (× 10^–6^ M)	%Found^a^	% Found^a^
	1.99	1.98	99.50	99.20
	3.98	3.97	99.77	100.73
	15.70	15.78	100.50	99.86
	31.00	30.98	99.92	
	97.50	97.42	99.92	
Mean			99.92	99.93
± S.D.			0.37	0.77
%RSD			0.37	0.77
*t* ^b^	0.01 (2.77)^b^			
*F*^b^	2.20 (19.00)^b^			

^a^Average of 3 replicate determinations.

^b^The values between parentheses are the tabulated values of *t* and *F* at *P* = 0.05^47^.

**Table 6:** Application of the proposed method to determining NTX to food samples.

Matrices	Bovine meat			Bovine kidney			Bovine fat			Bovine milk		
Parameter	Conc. taken (× 10^−6^ M)	Conc. found (× 10^−6^ M)	%Found^a^	Conc. taken (× 10^−6^ M)	Conc. found (× 10^−6^ M)	%Found^a^	Conc. taken (× 10^−6^ M)	Conc. found (× 10^−6^ M)	%Found^a^	Conc. taken (× 10^−6^ M)	Conc. found (× 10^−6^ M)	%Found^a^
	0.33	0.32	98.18	0.43	0.43	98.84	0.44	0.45	101.82	0.33	0.34	103.03
	0.43	0.43	99.77	0.53	0.53	100.76	0.52	0.51	97.88	0.43	0.44	103.40
	0.53	0.53	99.81	0.61	0.62	100.98	0.66	0.66	100.45	0.52	0.53	102.60
	0.61	0.63	103.76	0.71	0.71	99.30	0.80	0.80	100.00	1.82	1.82	100.72
Mean			99.89			99.97			100.04			102.43
± S.D			2.34			1.06			1.63			1.03
%RSD			2.34			1.06			1.63			1.00

^a^Average of 3 replicate determinations.

### Interference effect of commonly co-administered drugs

The potential interference likely to be introduced from commonly co-administered drugs was studied on the determination of NTX at 2.0 × 10^−5^ M. A systematic study of interference caused by each of: mebendazole, albendazole, flubendazole, cefotaxime and ivermectin was carried out. The peak potential of NTX was at 1.25 V, while the peak potentials for mebendazole, albendazole, flubendazole, cefotaxime were 1.1, 1.05, 1.07, 0.925 V, respectively, and no peak at all in case of ivermectin. The analysis of the obtained responses, revealed that these co-administered drugs did not interfere with the proposed approach, being far from the peak potential of the drug.

### Application to pharmaceutical preparation and food products

#### Pharmaceutical preparation

**Figure 5:**
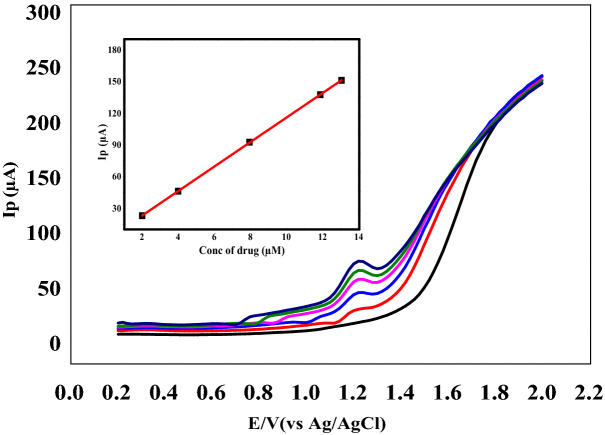
Square-wave voltammograms of different concentrations of PIONIX Vail (3.9 × 10^–6^–1.0 × 10^−4^ M) in Britton Robinson buffer (pH 3) at scan rates 100 mV s^−1^, using carbon paste electrode.

The proposed method was utilized successfully to assay NTX in its commercial vials, as shown in Fig. 5. The results were statistically compared with the official method using the t-test and F-test. The results were in good agreement with those obtained from the official method, as shown in Table 5.

#### Application to food samples

The determination of NTX residues in bovine meat, kidney, and fat samples was analyzed using the developed method. Before measurements, methanol was utilized for protein precipitation in all tissue samples. The applied method gave satisfactory results for determining NTX in bovine meat, kidney, fat, and milk, with recoveries for NTX in the range of 98.18–103.76, 98.84–99.3, 101.82–100.00, and 100.72–103.40%, respectively as shown in Table 6. These results confirm that NTX residues can be recognized by the developed method according to the European Union MRLs for each sample.

## Conclusion

The present work describes an accurate, simple, and sensitive sensor CPE for investigating NTX by SWV in its dosage form. It exhibit satisfactory detection and quantitation limits of 3.1 × 10^–7^ and 9.4 × 0^–7^ M, respectively. The analytical procedure was validated regarding linearity, precision, accuracy, LOD, LOQ, robustness and specificity. The proposed method was successfully extended for residual determination of NTX in bovine meat, kidney, fat, and milk samples.

## Data Availability

All data generated or analyzed during this study are included in this published article.
